# Cepharanthine as a Potential Novel Tumor-Regional Therapy in Treating Cutaneous Melanoma: Altering the Expression of Cathepsin B, Tumor Suppressor Genes and Autophagy-Related Proteins

**DOI:** 10.3389/fbioe.2020.601969

**Published:** 2020-12-01

**Authors:** Yufang Liu, Yang Xie, Yao Lin, Qingfang Xu, Yunfen Huang, Mengran Peng, Wei Lai, Yue Zheng

**Affiliations:** Department of Dermatology and Venereology, The Third Affiliated Hospital, Sun Yat-sen University, Guangzhou, China

**Keywords:** melanoma, cepharanthine, skin, cathepsin B, autophagy, treatment

## Abstract

The incidence of primary cutaneous melanoma continues to increase annually and is one of the most aggressive malignancies in humans and need to develop more novel non-surgical therapies. Autophagy and cathepsin B targeted therapy was reported to improve melanoma treatment. Cepharanthine (CEP), a natural alkaloid extracted from the genus Cephalophyllum has been reported to have the function of inhibiting cancers. We found that CEP inhibited human primary cutaneous melanoma cells viability and proliferation in 24 h *in vitro*, and topical application or intra-tumoral injection of CEP decreased the growth of cutaneous melanoma in mice within 4 weeks. CEP preparations below 50% concentration did not induce skin irritation and allergy reaction on human skin *in vivo*. Primary cutaneous melanoma cells incubated with CEP, the expression of cathepsin B was decreased and the LC3-I and LC3-II expression changed in a dose-dependent manner, while p53, p21Cip1p, and p16Inka gene expression was up-regulated. We demonstrated the effects of CEP as a novel tumor-regional therapy for cutaneous melanoma and provided a preliminary research basis for future clinical treatment researches and the exploration of integrated treatments with systemic therapy, radiotherapy, and surgery for human primary cutaneous melanoma.

## Introduction

The incidence of primary cutaneous melanoma continues to increase annually and is one of the most aggressive malignancies in humans. Primary cutaneous melanomas arise from melanocytes and are insensitive to conventional radiotherapy and systemic chemotherapy. The treatment of primary cutaneous melanoma included surgical excision (such as Mohs micrographic surgery) and non-surgical treatments (such as topical imiquimod and radiation therapy). Surgical treatment has problems such as unclear resection boundaries and unclean tumor resection leading to recurrence. So far, the most appropriate treatment time for PD-1/PD-L1 or MAPK inhibition is unclear, and its chronic cutaneous and systemic toxicity may receive more attention (Swetter et al., 2019). More novel non-surgical therapies need to be developed. Therefore, there is a need to investigate and develop more effective and less side-effect therapies and formulations for primary cutaneous melanoma treatment.

In recent years, targeted autophagy has been used as a potential therapeutic approach for melanoma treatment (Liu et al., 2013). Autophagy has been found to both promote and inhibit melanoma cell growth. Autophagy is a catabolic process that involves aging or impaired double-membrane sequestration and lysosomal breakdown of the cytoplasm. Studies have shown that autophagy plays an important role in the development of tumors, such as melanoma and breast cancer. Autophagy is both a cell survival pathway and a tumor suppressor pathway. The potential tumor suppressor function of autophagy in the initiation of tumor formation was discovered by Beclin 1 down-regulation and high p62 levels in cutaneous metastatic melanoma early stage, while increasing the expression LC3-II and decreasing the expression of p62 in the disease advanced stage (Corazzari et al., 2013).

Autophagy inhibition seems to be associated to, as indicated by Beclin 1 down-regulation and high p62 levels, while high number of autophagosomes, high levels of LC3-II and low levels of p62, indicating an increased level of autophagic activity, have been reported in patients in which cutaneous metastatic melanoma has become established.

Cathepsin B is thought to be involved in the diagnosis, treatment and prognosis of malignant tumors (Gong et al., 2013; Ruan et al., 2015). Studies have shown that targeting cathepsin B in combination with other relevant oncogenic molecules has significant therapeutic potential (Ruan et al., 2015). High cathepsin B expression is found in a variety of human cancers. Cathepsin B, a member of the cysteine protease family, hydrolyzes various extracellular matrix components and disrupts their mucosal barrier in pathological conditions. In recent years, cathepsin B has been considered a sword in the evolution of cancer (Mijanović et al., 2019). Aberrant regulation of cathepsin B expression correlates with the invasive and metastatic phenotype of cancer (Tomita et al., 1967). Cathepsin B can directly degrade or activate plasminogen activator and matrix metalloproteinases, and indirectly degrade many extracellular matrix and basement membrane components, such as laminin, fibronectin and type IV collagen, to promote the occurrence, development, invasion and distant transformation of tumors. Increasing the expression of cathepsin B in cancer cells can enhance the metastatic ability of cancer cells. Interference with RNA transcription of cathepsin B reduces tumor aggressiveness. Although progress has been made in the study of cathepsin B as a target for cancer gene therapy in combination with conventional chemotherapeutic agents, the role of cathepsin B in the proliferation of human primary cutaneous melanoma and its possible biological mechanism remain unclear to date.

**FIGURE 1:**
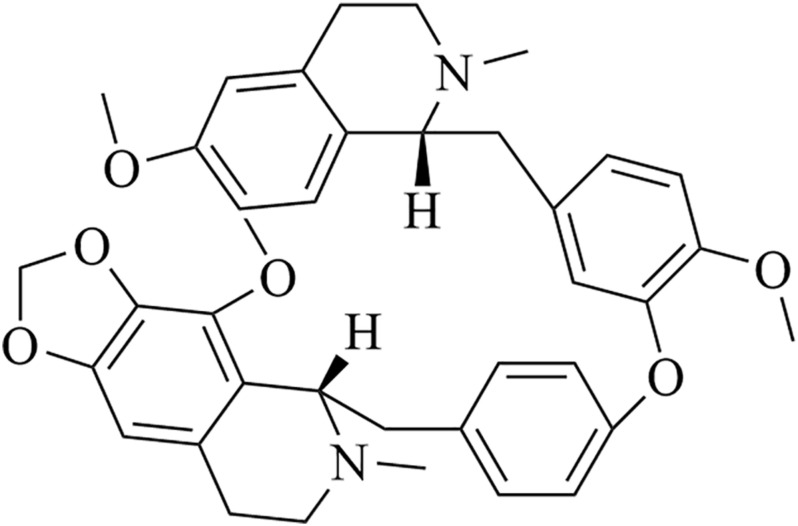
Chemical structure of CEP.

Cepharanthine (CEP, Figure 1), a natural alkaloid extracted from the genus Cephalophyllum and has been reported to have cathepsin B inhibitory function and to induce autophagy-related cell death in several cancer cells (Mijanović et al., 2019). CEP has been explored in many diseases such as cancer, alopecia areata, snakebite, and malaria (Saito et al., 1989). In recent studies, it was identified that CEP may potently induce apoptosis in murine leukemia cell lines, adenosquamous carcinoma cell lines, and OSCC cell lines (Furusawa et al., 1998; Harada et al., 2001; Harada et al., 2003). It has been reported that CEP not only exerts antitumor effects by improving the immune activity of the host (Morioka et al., 1985; Ebina and Murata, 1991), but also can increase the effects of chemotherapeutic agents, such as anticancer agents of adriamycin and doxorubicin (Asaumi et al., 1995; Hotta et al., 1997; Lyu et al., 2017; Bailly, 2019). However, its potential anticancer effect in skin melanoma has not been clarified yet, especially in skin regional usage.

This study investigated the effect of CEP as a novel tumor-regional treatment for the cutaneous melanoma and explored its mechanism in inhibiting the human primary cutaneous melanoma cells.

## Materials and Methods

### Reagents

Cepharanthine was purchased from Shiji-Aoke (Beijing, China), cell counting kit-8 was bought from APExBio (Houston, TX, United States), DMEM, penicillin, streptomycin, fetal bovine serum (FBS) and PBS were obtained from Gibco Life Technologies (Grand Island, NY, United States). Anti-GAPDH, P62 and anti-rabbit IgG HRP-linked antibody were obtained from Cell Signaling Technology, Inc., (Beverly, MA, United States). Cathepsin B antibody was purchased from the Santa Cruz Biotechnology (Santa Cruz, CA, United States). Primary antibodies against LC3 were obtained from Abcam (Cambridge, MA, United States), anti-rabbit HRP-conjugated secondary antibody and anti-mouse HRP-conjugated secondary antibody were purchased from Cell Signaling Technology (Boston, MA, United States).

### Ethics Statement

The human studies were approved by the Clinical Research Ethics Committee at the Third Affiliated Hospital of Sun Yat-sen University, Guangzhou, China (No: [2015]2-107). The consent procedure was conducted according to the principles expressed in the Declaration of Helsinki. All subjects read and signed the informed consent.

### Cell Culture

Human primary cutaneous melanoma cells were derived from five primary cutaneous melanoma patients. Patients with primary cutaneous melanoma were diagnosed at the Third Affiliated Hospital of Sun Yat-sen University. Resected tumor from the patients was dissociated with mechanical dissection followed by enzymatic digestion. Tumor mononuclear cells (TMCs) were then isolated by Ficoll-Paque PLUS centrifugation, washed in complete Dulbecco’s modified Eagle’s media, and used fresh. All sample collection and experimental procedures were approved by the Ethics Board of the Third Affiliated Hospital of Sun Yat-sen University, Guangzhou, China. Written consent form was obtained from each participant. Human melanoma cells were cultured Dulbecco’s modified Eagle medium (DMEM, Gibco, Grand Island, NY, United States) supplemented with 100 U/mL penicillin/streptomycin and 10% fetal bovine serum (FBS; Gibco) 100 unit/ml penicillin at 37°C in a humidified atmosphere containing 5% CO_2_.

Human epidermal melanocytes isolated from circumcised foreskins were cultured in Medium 254 (M254 cat. no. M-254-500 Gibco, United States) supplemented with 100 U/mL penicillin/streptomycin, 10% fetal bovine serum (FBS; Gibco), and 5 ml Human Melanocyte Growth Supplement (HMGS, cat. no. S-002-5). They were grown at 37°C in a humidified 5% CO_2_ atmosphere and used for experiments up to passage number ten. Repeat experiments were performed using cells from different donors.

### Skin Melanocytes and Melanoma Cells Incubated With Cathepsin B

The proportions of apoptotic melanocytes, melanoma cells and melanoma cell cultured with 200 ug/L cathepsin B for 24 h. Melanoma cells were divided into six groups, added different concentration of cathepsin B, including 0, 12, 25, 50, 100, and 200 ug/L in each well.

### CEP Added in Human Melanoma Cells

Cells were incubated in 96-well culture plates overnight, and then treated with concentrations of CEP of 1.25, 2.5, 5, 10, 20, and 40 mg/L in each well. After incubation in fresh culture medium for 24, 48, 72 h, cells were washed three times with PBS.

### MTT

Human epidermal melanocytes and melanoma cells cultured with cathepsin B (200 ug/L) for 24 h were detected by 3-(4,5)-dimethylthiahiazo(-z-y1)-3,5-di-phenytetrazoliumromide (MTT) MTT and flow cytometry. Human primary cutaneous melanoma cells were cultured in triplicate in a 96-well plate (4 × 103 per well), and the plates were placed in incubation box at 37°C with 5% CO_2_. The cells were treated either with various concentrations (0, 12.5, 25, 50 100, and 200 ug/L) of cathepsin B or without cathepsin B and after cells attached to the culture plate 50 min and 24-hour culture, MTT assay (Gibco, United States) was performed to determine the cell apoptosis. The culture media were placed and 10 μl MTT assay (5 mg/ml) was added to each well after cells attached to the culture plate 50 min. After 10 min oscillation, the optical density (OD) values were measured with a microplate reader (Beckmann Coulters) at 490 nm, sign 0H. Then the culture media were placed and 10 μl MTT assay (5 mg/ml) was added to each well after 24 h incubation. After 10 min oscillation, the OD values were measured with a microplate reader (Beckmann Coulters) at 490 nm, sign 24H. Cell viability was presented as a percent of MTT reduction in the treated cells versus the controls (cells incubated in cathepsin B-free medium). The relative MTT level (%) was calculated as [A]/[B] × 100%, where [A] is the absorbance of the test sample and [B] is the absorbance of control sample containing the untreated cells. Decreased relative MTT level indicates decreased cell viability. This experiment was performed in triplicate, and the statistical analysis was performed to obtain the final values.

### CCK-8

After 24 h, cellular viability was detected with Cell Counting Kit-8 (CCK-8) assay. Human primary cutaneous melanoma cells were cultured in triplicate in a 96-well plate (4 × 103 per well), and the plates were placed in incubation box at 37°C with 5% CO_2_. The cells were treated either with various concentrations (1.5, 2.5, 5, 10, 20, and 40 mg/L) of CEP or without CEP. Cellular viability and proliferation were detected with CCK-8 test kit (Dojindo, Japan) assay according to instructions. It determines cell viability and proliferation by detecting dehydrogenase activity in living cells. The OD value of the formazan dye produced by dehydrogenase to absorb a wavelength of 450 nm reflects the content of formazan dye, and it is proportional to the number of viable cells.

### Flow Cytometry Analysis

At 24 h after CEP addition, all four groups of cells were fixed with 70% ethanol for 12–16 h at 4–6°C and then incubated with 1 mg/ml RNase R (Sigma-Aldrich, St. Louis, MI, United States) for 0.5 h at 37°C. DNA was labeled using propidium iodide (Sigma-Aldrich, St. Louis, MI, United States) at a concentration of 50 mg/ml for 0.5 h at 4–6°C. DNA content was assessed by flow cytometry (Beckman Coulter, High Wycombe, United Kingdom) and results were analyzed by Modfit LT v3.2 software.

### Western Blot

Total cellular protein was extracted with the protein extraction kit (KeyGen Biotech, Nanjing, China). The modified Lowry protein assay kit (Pierce, Rockford, IL, United States) was used to quantify protein. Equal amounts of protein were analyzed using 10% SDS–PAGE, and transferred to a PVDF membrane. PVDF membranes were incubated with primary rabbit anti-LC3B (CST, 3868S,1:1200), primary mouse anti-cathepsin B (ab58802,1:1000), rabbit anti-P53 (ab131442,1:1000), rabbit anti-p21Cip1p (CST,2947S,1:1000), rabbit anti-p16INKa (CST,80772S,1:1000), rabbit anti-P62 (CST,16177S,1:1000), and anti-GAPDH (ab181602,1:2000) incubated overnight at 4°C. Following subsequent incubation with anti-rabbit HRP-conjugated secondary antibody (Affinity, S0001, 1:2000) or anti-mouse HRP-conjugated secondary antibody (Affinity, S0002, 1:2000), blots were visualized with enhance chemiluminescence (Millipore, Billerica, MA, United States). Bands on X-ray film were scanned with GS-800 Calibrated Densitometer (Bio-Rad, Hercules, CA, United States). The intensity of bands was quantified with Quantity one software. All values were normalized to the corresponding GAPDH.

### Nude Mice and Tumor Inoculations

All animal studies were ensured to complied with the ARRIVE guidelines. The protocol was approved by the Committee on the Ethics of Animal Experiments of the Third Affiliated Hospital of Sun Yat-sen University. A total of 30 male Bal/bc nude mice (Jiangsu Jicui Yaokang Biotechnology Co., China) purchased at 3 weeks of age with 18–20 g weight. The mice were provided with sterile water and food. inside a laminar flow hood. The melanoma model was established in the nude mice via cells injection: A-375 melanoma cells (1 × 10^7^) were suspended in 0.1 ml serum-free medium and injected into the subcutaneous tissue of 4-week-old nude mice. Tumors were allowed to grow for 7 days before CEP treatment. The mice were then divided into 3 groups, each of 10 mice with similar mean tumor volumes (between 60 and 65 mm^3^). At the study end points, mice were sacrificed by cervical dislocation. The present study was performed following the recommendations from the Institutional Animal Care and Use Committee of the Third Affiliated Hospital of Sun Yat-sen University.

### *In vivo* CEP Treatment Protocol

When solid tumors grew to 40–50 mm^3^, the mice were then divided into 3 groups, each of 10 mice: group A: melanoma mice without any treatment (control); group B: 5 ml (4000 mg/L with PBS solvent) CEP was dosed on cotton piece and then topically fixed on the tumor skin with T.R.U.E. TEST^®^ patch test system (True Test Technology Inc., Mineral Wells, TX, United States) which was cut into small single pieces, 5 times/week for 4 weeks. The cotton piece contained the same CEP concentration was changed every day; and group C: 20 mg/kg CEP dissolved in PBS solvent was injected into the melanoma mass (Harada et al., 2009), 5 times/week for 4 weeks. The tumors were measured every 7 days and the relative tumor volumes were calculated. At the time point of 28th day, mice were sacrificed by cervical dislocation.

### Patch Test on Human Body

Thirty-two healthy Chinese subjects (15 males, 15 females; mean age 30–50 years) completed patch testing. The subjects were healthy and had fair skin with no significant skin disease or known history of atopic dermatitis. None of the subjects had any dermatological problems and no history of drug hypersensitivity or abnormal reactions to sunlight. They had not taken medication for 1 month and had not received back phototherapy for 6 months prior to the study. Subjects were instructed to avoid medication and back sun exposure throughout the study.

The 5, 10, 25, and 50% CEP preparations were applied separately to the subject’s skin using the T.R.U.E. TEST^®^ Patch Testing System (True TEST Technology Inc., United States). In the small plate, CEP was kept in direct contact with the upper back skin for 48 h. Tape them in place and mark the test site. Subjects were instructed not to wash the area or perform strenuous exercise during the 48 h of patch retention. After that, the patch was removed and a preliminary reading was taken 1 h later. Reactions were read at 24, 48, and 72 h after removal.

Path testing reaction was read as follows:

• – Negative
• ? Doubtful reaction: faint macular erythema only
• + Weak (non-vesicular) positive reaction: erythema, infiltration, possibly papules
• + + Strong (vesicular) positive reaction: erythema, infiltration, papules, vesicle
• + + + Extreme positive reaction: bullous reaction
• IR Irritant reaction of different types: pustules as well as patchy follicular or homogeneous erythema without infiltrations are usually signs of irritation and do not indicate allergy.

### Statistical Analysis

Data were presented as the mean ± S.E. Statistical analysis for comparison of two groups, included the dates of MTT, CCK-8, flow cytometry analysis, tumor volume and tumor weight, was subject to a two-tailed student’s *t*-test. For comparison of multiple groups, included the dates of MTT, CCK-8, flow cytometry analysis, tumor volume, and tumor weight, one-way analysis of variance (ANOVA) followed by S-N-K *post hoc* test was performed.

*P*-value of <0.05 was considered statistically significant. All experiments were performed twice and data analysis was evaluated using SPSS Version 13.0 software.

## Results

### Apoptosis Rate of Melanocytes and Cutaneous Melanoma Cells Treated With Cathepsin B

**FIGURE 2:**
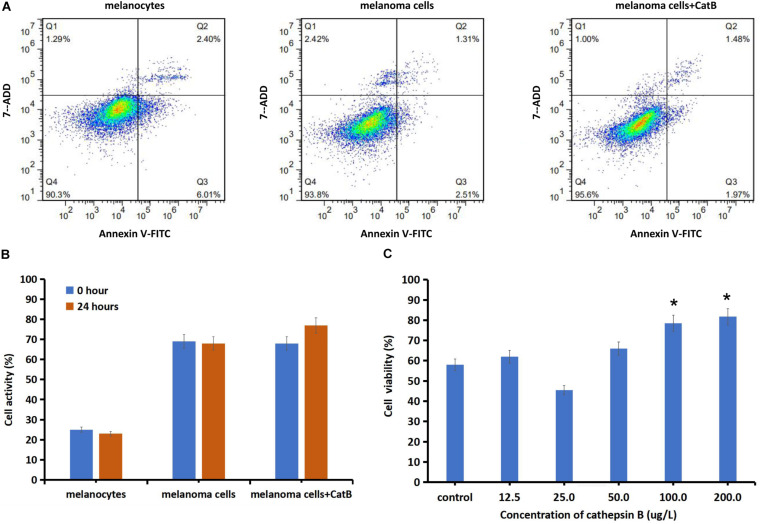
Changes of cell activity and viability of normal skin melanocytes and melanoma cells by exogenously adding cathepsin B. **(A)** The proportions of apoptotic melanocytes, melanoma cells and melanoma cell cultured with cathepsin B (200 ug/L, 24 h) were detected using flow cytometry. After adding cathepsin B, the total apoptotic value of melanoma cells was decreased from 3.82 to 3.45% (*P* < 0.05). **(B)** Cellular activity was detected with MMT assay. The cellular activity of melanoma cells was increased compared to the normal melanocytes. After added 200 ug/L cathepsin B for 24 h, melanoma cells activity was increased 12.7% compared with the control melanoma cells (*P* < 0.05). **(C)** Cellular viability was detected with cck-8 assay. After added different concentrations of cathepsin B of 12.5, 25, 50, 100, and 200 ug/L in melanoma cells, the cell viability was increased at the dose of 100 and 200 ug/L (*P* < 0.05, compared to the control). *P < 0.05.

The apoptosis rate of primary melanoma cells was 3.82 ± 0.14%, which was lower than the melanocytes (8.41 ± 1.25%) (*P* = 0.012). Melanoma cells incubated with 200 ug/L cathepsin B for 24 h, the total apoptotic value of melanoma cells was decreased from 3.82 ± 0.12 to 3.45 ± 0.09% (*P* = 0.03) (Figure 2A). Melanoma cells incubated with 200 ug/L cathepsin B for 24 h, the cellular activity was increased 12.7 ± 1.59% comparing with the control (*P* = 0.015) (Figure 2B). Primary cutaneous melanoma cells incubated with cathepsin B for 24 h, the cell apoptosis rate was increased in a dose-dependent manner. After adding different concentration of cathepsin B (12.5, 25, 50, 100, and 200 ug/L) in melanoma cells for 24 h, the cell viability was significantly increased at the dose of 100 and 200 ug/L(*P* < 0.05, compared to the control) (Figure 2C).

### Cells Proliferation Rate of Human Melanoma Cells Incubated With CEP

**FIGURE 3:**
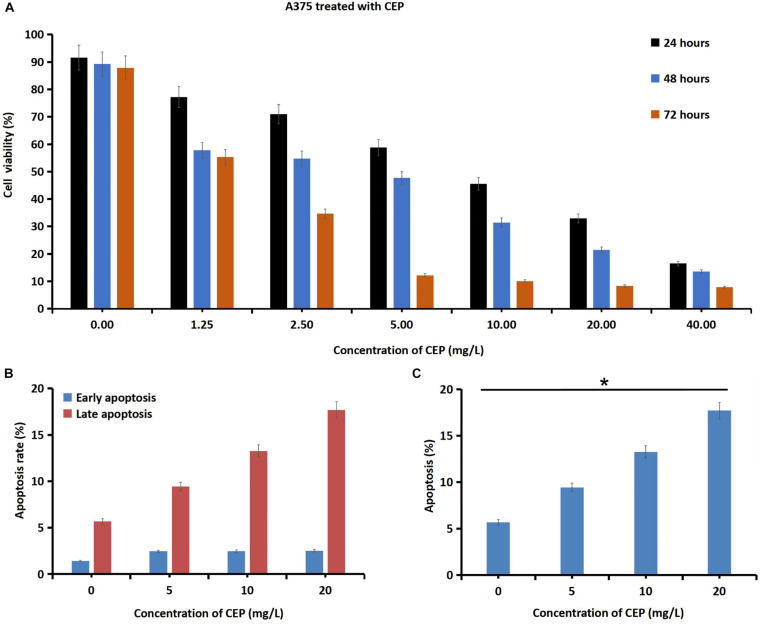
CEP inhibited human melanoma cells proliferation viability and increased cell apoptosis rates. **(A)** Human melanoma cells were incubated with 1.25L, 2.5, 5, 10, 20, and 40 mg/L CEP for 24, 48, 72 h. Then the cells proliferation rate was detected via CCK-8 method. The data showed that the proliferation viability of human melanoma cells was decreased in a dose-dependent manner. Melanoma cells incubated with 40 mg/L CEP, the cell proliferation rate was decreased from 87.84 ± 7.9% (0 h, control) to 16.48 ± 9.4% after 24 h, to 13.52 ± 5.3% after 48 h and to 7.8 ± 2.9% after 72 h. **(B)** After melanoma cells were treated with CEP for 24 h, the cell apoptosis rate was tested via Flow cytometry analysis and showed a concentration-dependent increasing. The early apoptosis rate was 2.463 ± 0.231% (5 mg/L CEP), 2.477 ± 0.067% (10 mg/L CEP), 2.500 ± 0.642% (20 mg/L CEP), which was increased comparing with the control (1.420 ± 0.105%; *P* = 0.0380). **(C)** The late apoptosis rate was 9.433 ± 0.071% (5 mg/L CEP), 13.267 ± 0.065% (10 mg/L CEP), 17.700 ± 0.243% (20 mg/L CEP) which was increased comparing with the control (5.673 ± 0.195%; *P* < 0.01). *P < 0.05.

After melanoma cells were treated with CEP for 24, 48, and 72 h, the cells proliferation rates were decreased in a dose-dependent manner (Figure 3A). CEP at 40 mg/L inhibited human primary cutaneous melanoma cells by 87.84 ± 7.9% (0-hour, control), 16.48 ± 9.4% (24-hour), 13.52 ± 5.3% (48-hour) and 7.8 ± 2.9% (72-hour), respectively. Melanoma cells were treated with CEP for 24 h, the cell apoptosis rate increased in a dose-dependent manner (Figure 3B). The late apoptosis rates were increased in a dose-dependent manner as well (Figure 3C). After melanoma cells were incubated with CEP for 24 h, the early apoptosis rates were 2.463 ± 0.231% (5 mg/L CEP), 2.477 ± 0.067% (10 mg/L CEP), 2.500 ± 0.642% (20 mg/L CEP), and increased compared with the control (1.420 ± 0.105%; *P* = 0.0380, and the late apoptosis rates were 9.433 ± 0.071% (5 mg/L CEP), 13.267 ± 0.065% (10 mg/L CEP), and 17.700 ± 0.243% (20 mg/L CEP), respectively, compared with control group (5.673 ± 0.195%, *P* < 0.01).

### Cathepsin B Expression Changes After Adding CEP

**FIGURE 4:**
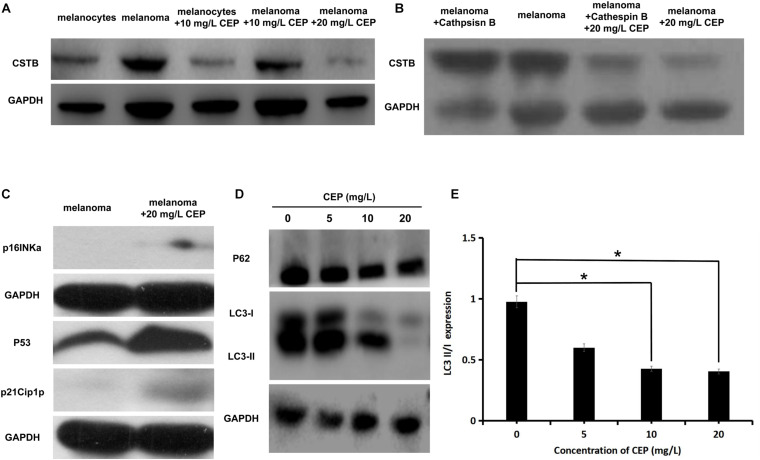
CEP inhibited cathepsin B and autophagy while activated tumor suppressor gene-related proteins in human melanoma cells. **(A)** CEP inhibited cathepsin B in melanoma cells. Cathepsin B protein quantification of the 10 mg/L CEP treated cells via Western blot. The Cathepsin B expression was decreased after 10 mg/L CEP treatment for 24 h. The quantification of protein gray value was decreased from 0.893 ± 0.018 (control group) to 0.779 ± 0.018 (CEP group, *P* = 0.027). **(B)** Overexpressing cathepsin B did not lead to inhibition of CEP activity, 20 mg/L CEP could also decrease cathepsin B overexpressed in melanoma cells. **(C)** CEP active tumor suppressor gene-related proteins expression in melanoma cells. Cell lysates were analyzed by western blot with anti-P53, anti-p21Cip1p, and anti-p16INKa antibodies, respectively. P53, p21Cip1p and p16INKa expression was increased after adding 20 mg/L CEP for 72 h. **(D)** The western blot analysis of P62, LC3-I and LC3-II. Protein quantification showed that after treating with 5 mg/L, 10 mg/L, 20 mg/L CEP for 24 h, P62 expression in human melanoma cells had no significate change (*P* > 0.05). **(E)** After treating with 5 mg/L, 10 mg/L, 20 mg/L CEP for 24 h, autophagy-related protein LC3-I and LC3-II expression was quantified via western blot which showed decreased in dose-dependent manner. LC3II/I value was decreased from 0.975 ± 0.096 (0 mg/L CEP) to 0.600 ± 0.082 (5 mg/L CEP, *P* > 0.05), 0.427 ± 0.046 (10 mg/L CEP, *P* < 0.05), 0.405 ± 0.086 (20 mg/L CEP, *P* < 0.05), respectively. *P < 0.05.

Incubated with 10 mg/L CEP for 24 h, the expression of cathepsin B was decreased by 32.12 ± 7.92% in melanocytes and 10.48 ± 1.23% in melanoma cells. Incubated with 20 mg/L CEP for 24 h the expression of cathepsin B was decreased by 67.67 ± 8.17% in melanoma cells (Figure 4A). Overexpressing cathepsin B did not lead to inhibition of CEP activity, 20 mg/L CEP could also decrease cathepsin B overexpressed in melanoma cells (Figure 4B).

### Antioncogene Expression Changes

Western blot showed that P53, p21Cip1p and p16INKa expression was increased in human melanoma cells after incubating with 20 mg/L CEP for 72 h (Figure 4C). 5, 10, and 20 mg/L CEP incubated with melanoma cells for 24 h, P62 expression in human melanoma cells had no significate change (*P* > 0.05) (Figure 3D).

### Autophagy-Related Protein Expression

A total of 5, 10, 20 mg/L CEP incubated with melanoma cells for 24 h, autophagy-related protein LC3-I and LC3-II was decreased in Figure 4D. Incubation of melanoma cells with different concentrations of CEP for 24 h decreased the expression of autophagy-related proteins LC3-I and LC3-II in a dose-dependent manner, with LC3II/I values varying as 0.975 ± 0.096 (0 mg/L CEP), 0.600 ± 0.082 (5 mg/L CEP, *P* > 0.05), 0.427 ± 0.046 (10 mg/L CEP, *P* < 0.05), 0.405 ± 0.086 (20 mg/L CEP, *P* < 0.05) (Figure 4E), respectively.

### Effects of CEP on Melanoma Growth *in vivo*

**FIGURE 5:**
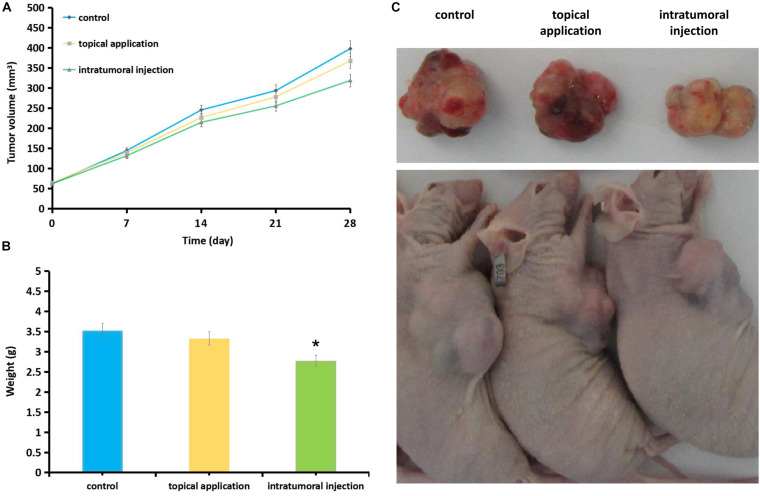
Effect of CEP on melanoma tumor growth in nude mice. Mice were treated with CEP for 4 weeks (5 times/week) and tumor growth was measured every 7 days during the treatment period. At 28 days, mice were sacrificed by cervical dislocation and the tumor mass was take out and weighed. **(A)** Intra-tumoral inject CEP (20 mg/kg), the tumor volume was 214.36 ± 12.46 mm^3^ (*P* = 0.069) at 14-day, 255.25 ± 7.85 mm^3^ (*P* = 0.042) at 21-day and 318.69 ± 12.98 mm^3^ (*P* = 0.031) at 28-day compared with the control in the same period as 245.22 + 10.63, 293.27 + 9.61, and 397.86 ± 15.57 mm^3^, statistically significant growth inhibition was found by comparing day-21 and day-28 dates in intra-tumoral inject CEP groups. Topically application of 5 ml (4000 mg/L with PBS solvent) CEP for 28 days the tumor volume was 367.46 ± 11.57 mm^3^ (*P* = 0.042) compared with 397.86 ± 15.57 mm^3^ as control, which also showed the inhibition of tumor growth but the effects were weaker than that of the intra-tumoral injection group. **(B)** At 28 days the tumor weight was: 3.331 ± 1.84 g of CEP topical application group, 2.776 ± 1.29 g of CEP intra-tumoral injection group and 3.523 ± 2.01 g of control group. Significant different was found comparing intra-tumoral injection group (*P* = 0.042) and control group, while no significant different between CEP topical application group (*P* = 0.072) and the control group. Data are presented as the mean ± standard deviation from three separate experiments. Each group contained 10 mice. *P < 0.05 vs. control. **(C)** The gross inspection of the sacrificed mice and the tumors.

At 28 days, mice were sacrificed by cervical dislocation and the tumor mass was take out and weighed (Figure 5A). After intra-tumoral injecting of CEP (20 mg/kg), the tumor volume was decreased as 214.36 ± 12.46 mm^3^ (*P* = 0.069) at 14-day, 255.25 ± 7.85 mm^3^ (*P* = 0.042) at 21-day and 318.69 ± 12.98 mm^3^ (*P* = 0.031)at 28-day comparing with the control in the same period as 245.22 + 10.63, 293.27 + 9.61, and 397.86 ± 15.57 mm^3^. Statistically significant growth inhibition was found with intra-tumoral injection CEP at day-21 and day-28. Topically applying 5 ml (4000 mg/L) CEP for 28 days, the tumor volume was 367.46 ± 11.57 mm^3^ smaller than the control (397.86 ± 15.57 mm^3^, *P* = 0.042) (Figure 5B).

At 28 days the tumor weight was 3.331 ± 1.84 g of CEP topical application group, 2.776 ± 1.29 g of intra-tumoral injection group and 3.523 ± 2.01 g of control group. Significant different was found comparing intra-tumoral injection group and control group (*P* = 0.042), while no significant different between topical application group and control group (*P* = 0.072) (Figure 5C).

### Safety Evaluation of CEP on Human Body Skin

**FIGURE 6:**
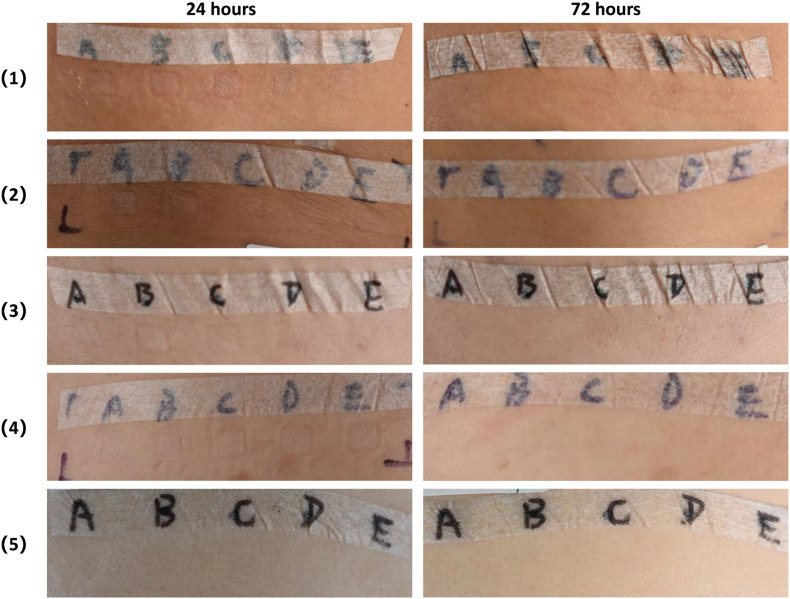
Safety evaluation of CEP on human body skin via closed patch test. CEP solutions at the concentration of 5% (A), 10% (B), 25% (C), 50% (D), control solution (E) was applied separately on the subjects’ skin using the T.R.U.E. TEST patch test system. After 48 h, the patches were removed and an initial reading was taken 1 h later. Reaction reading was carried out 24 and 72 h after removing. At 24- and 72-hour readings, 5, 10, 25, 50% CEP solution did not induce skin irritation and allergy reaction in 32 subjects’ skin.

After 5, 10, 25, 50% CEP preparations were applied separately on human skin for 72 h, the paths ware removed and the skin reaction was read at 24- and 72-hour. 5, 10, 25, 50% CEP preparations did not induce skin irritation and allergy reaction in 32 subjects’ skin (Figure 6).

## Discussion

Cepharanthine (CEP) exhibiting multiple pharmacological properties including anti-oxidative, anti-inflammatory, immuno-regulatory, anti-cancer, anti-viral and anti-parasitic, was selected as a candidate for investigating the local treatment effects of tumor primary cutaneous melanoma. But the multiple antitumor effects of CEP including the molecular biology and genetic cross-talk mechanisms in primary cutaneous melanoma cells still need further identification.

The interaction of molecular biology and genetics plays an important role in melanocyte malignant transformation and melanocyte damage. Malignant tumors can produce a variety of hydrolytic enzymes, degrade the extracellular matrix, disrupt the host mucosal barrier, and promote tumor cell invasion through the basement membrane and distant metastasis.

High expression of cathepsin B has been detected in a variety of tumor tissues including primary cutaneous melanoma and the serum of tumor patients (Fröhlich et al., 2001) and can be used as one of the melanoma serum biomarkers (Zhang et al., 2011). Cathepsin B regulates the production/signaling of TGF-β and promotes fibroblast activation which in turn promotes invasive growth of melanoma cells (Girotti et al., 2011). It is also regulated by the collagen I/α2β1 integrin axis and the non-receptor tyrosine kinases Abl and Arg (Abl/Arg) -activated transcription factors (i.e., Ets1, Sp1, and NF-κB/p65) (Tripathi et al., 2018). Targeting cathepsin B has been found not only to reduce the development of malignant melanotic tumors (Payon et al., 2019), but also to inhibit tumor growth and metastasis in mice (Qifan et al., 2016).

Some studies have reported that CEP inhibits cathepsin B function by inhibiting the maturation of lysosomal cathepsin B and cathepsin D (Mijanović et al., 2019). Its anticancer effects have been studied in a variety of cancer cells, such as nasopharyngeal carcinoma cells, cholangiocarcinoma cells, and oral squamous cell carcinoma cells (Corazzari et al., 2013; Liu et al., 2013; Li et al., 2019b). CEP antitumor effects have been reported to lie in the inhibition of P-glycoprotein activity (Nakajima et al., 2004; Ikeda et al., 2005), or by modulating the expression of Bcl-2 and BAX proteins (Biswas et al., 2006; Kikukawa et al., 2008). In addition, others have found that CEP significantly increased the expression of p21Waf1 protein and decreased the expression of cyclins A and D proteins to exert the antitumor effect of ovarian cancer cells (Bellei et al., 2014). But CEP has been poorly studied in cutaneous melanoma, and in our study, it not only downregulates cathepsin B levels but also possesses novel antitumor mechanisms and inhibits primary cutaneous melanoma activity and metastasis by either topical application or intra-tumoral injection in mice.

Autophagy is a protective mechanism against chemotherapy-induced cell death in melanoma. Autophagy inhibition inhibits cancer initiation at an early stage, as indicated by downregulation of Beclin 1 and upregulation of p62 expression. Low levels of p62 and high levels of LC3-II, indicating increased levels of autophagy, promote skin metastasis. Modulation of autophagy is now being explored as a therapeutic option in melanoma treatment. It has been found that the “BRAF-TFEB-autophagy-lysosome” axis represents a key regulatory pathway in BRAF mutant melanoma, leading to tumor progression, metastasis, and resistance to BRAF-targeted therapy in melanoma (Li et al., 2019a; Barceló et al., 2020). Autophagy inhibitors [e.g., hydroxychloroquine (HCQ)] in combination with standard chemotherapy regimens synergistically increase cell death in melanoma (White and DiPaola, 2009). Studies have also found that cathepsin B-mediated proteolysis of DAB2, a tumor suppressor, induces cell entry into autophagy, promotes metastasis (Bhoopathi et al., 2010). Jiang et al. (2016) found that cathepsin B could regulate TGF-beta-induced autophagy by mediating the cleavage of disabled-2. These indicated that the molecular link of cathepsin B between autophagy and apoptosis in tumors, and suggested the role of targeting cathepsin B in cancer therapy (Ruan et al., 2015).

Autophagy targeted therapy could improve the treatment for metastatic melanoma. Gao et al. (2017) identified that cepharanthine could induce autophagy, apoptosis and cell cycle arrest in cancer cells. CEP has been found to induce autophagy-related cell death in several cancer cells, acting by blocking autophagosome-lysosome fusion and maturation of cathepsin B and cathepsin D (Tang et al., 2018; Mijanović et al., 2019), or by blocking the Akt/mTOR signaling pathway to induce autophagy and apoptosis in tumor cells. In this study, we found that the expression of the autophagy-related gene proteins LC3-I and S LC3-II was downregulated by CEP in primary cutaneous melanoma cells. This demonstrates that CEP can inhibit the growth of primary cutaneous melanoma by inhibiting autophagy and cathepsin B.

We found not only that cathepsin B and autophagy-related protein expression were decreased by CEP, but also that p53, p21Cip1p, and p16Inka expression were simultaneously increased in human melanoma cells. Topical application and intra-tumoral injection of CEP preparations could decrease the growth of primary cutaneous melanoma and did not induce irritation of human skin at certain concentrations. The mechanisms leading to malignant transformation of melanocytes and melanocytic lesions include complex endogenous factors, such as gene mutation, dysregulation of cell proliferation, abnormal autophagy, and molecular biological changes. Previous studies have shown that mutations in P53, P21, CDKN2A, A CDK4, CDKN2A, p16 (INK4A), p14, MC1R, and DNA repair genes predispose to melanoma (Bandarchi et al., 2013).

Previous studies have revealed that CEP could inhibit ABCC10 (also known as MRP7) which is a broad-specificity transporter of xenobiotics, including many antitumor drugs (taxanes, epothilone B, vinca alkaloids, cytarabine, tamoxifen) and accepted as one of the most important of drug efflux transporters overexpressed on the membrane of cancer cells majorly cause the multi-drug resistance to cancer chemotherapy (Kathawala et al., 2015).

Although we identified the CEP anti-tumor effects in mice via topical application or intra-tumoral injection, the *in vivo* antitumor efficacy of CEP, as monotherapy, was not obvious. Ikeda et al. revealed that CEP could potently enhance the sensitivity to cytotoxic agents such as doxorubicin in K562 leukemia cells, not only via a reversal of ABCB1- mediated multi-drug resistance (MDR) but also increasing the accumulation of doxorubicin in nuclei (Ikeda et al., 2005). In addition, we found that CEP preparations below 50% concentration did not induce skin irritation and allergy reaction on human skin *in vivo*. Thus, combination use of others drugs such as chemo drugs to improve or magnify cepharanthine’s efficacy *in vivo*, and integrated treatments of topical CEP with systemic therapy, radiotherapy, and surgery could be further explored in treating cutaneous melanoma. In the aspect of anti-tumor mechanism research, more comprehensive detection of differentially expressed genes is needed and further biosafety evaluation *in vivo* are need to better understanding the effectiveness and safety of CEP as a potential novel tumor-regional therapy in treating cutaneous melanoma.

## Conclusion

In this study, we demonstrated the effects of CEP as a novel tumor-regional therapy for cutaneous melanoma basing on the finding that CEP could inhibit the growth of human primary cutaneous melanoma cells by altering the expression of cathepsin B, tumor suppressor genes, and autophagy-related proteins. These findings provided a preliminary research basis for future clinical treatment researches and might also contribute to the further development of novel local antineoplastic agents and the exploration of integrated treatments with systemic therapy, radiotherapy, and surgery for human primary cutaneous melanoma.

## Data Availability Statement

The raw data supporting the conclusions of this article will be made available by the authors, without undue reservation.

## Ethics Statement

This study was approved by the Clinical Research Ethics Committee at the Third Affiliated Hospital of SunYat-sen University, Guangzhou, China (No: [2015]2-107). The consent procedure was conducted according to the principles expressed in the Declaration of Helsinki. All subjects read and signed the informed consent. The patients/participants provided their written informed consent to participate in this study. All animal studies were ensured to comply with the ARRIVE guidelines. The protocol was approved by the Committee on the Ethics of Animal Experiments of the Third Affiliated Hospital of Sun Yat-sen University. Both the consent procedure and our study were approved by the Clinical Research Ethics Committee and Animal Care and Use Committee (IACUC) of the Third Affiliated Hospital of Sun Yat-sen University, Guangzhou, China and followed the internationally recognized guidelines.

## Author Contributions

All authors listed have made a substantial, direct and intellectual contribution to the work, and approved it for publication.

## Conflict of Interest

The authors declare that the research was conducted in the absence of any commercial or financial relationships that could be construed as a potential conflict of interest.
